# NeuroDAC: an open-source arbitrary biosignal waveform generator

**DOI:** 10.1088/1741-2552/abc7f0

**Published:** 2021-02-05

**Authors:** M P Powell, J Anso, R Gilron, N R Provenza, A B Allawala, D D Sliva, K R Bijanki, D Oswalt, J Adkinson, N Pouratian, S A Sheth, W K Goodman, S R Jones, P A Starr, D A Borton

**Affiliations:** 1 School of Engineering, Brown University, Providence, RI, United States of America; 2 Carney Institute for Brain Science, Brown University, Providence, RI, United States of America; 3 VA RR&D Center for Neurorestoration and Neurotechnology, Providence VA Medical Center, Providence, RI, United States of America; 4 Department of Neuroscience, Brown University, Providence, RI, United States of America; 5 Department of Neurosurgery, University of California, San Francisco, San Francisco, CA, United States of America; 6 The Charles Stark Draper Laboratory, Inc., Cambridge, MA, United States of America; 7 Department of Neurosurgery, Baylor College of Medicine, Houston, TX, United States of America; 8 Menninger Department of Psychiatry and Behavioral Sciences, Baylor College of Medicine, Houston, TX, United States of America

**Keywords:** waveform generator, biosignal playback, closed-loop neuromodulation, neural interface, biomedical devices

## Abstract

**Objective.:**

Researchers are developing biomedical devices with embedded closed-loop algorithms for providing advanced adaptive therapies. As these devices become more capable and algorithms become more complex, tasked with integrating and interpreting multi-channel, multi-modal electrophysiological signals, there is a need for flexible bench-top testing and prototyping. We present a methodology for leveraging off-the-shelf audio equipment to construct a biosignal waveform generator capable of streaming pre-recorded biosignals from a host computer. By re-playing known, well-characterized, but physiologically relevant real-world biosignals into a device under test, researchers can evaluate their systems without the need for expensive *in vivo* experiments.

**Approach.:**

An open-source design based on the proposed methodology is described and validated, the NeuroDAC. NeuroDAC allows for 8 independent channels of biosignal playback using a simple, custom designed attenuation and buffering circuit. Applications can communicate with the device over a USB interface using standard audio drivers. On-board analog amplitude adjustment is used to maximize the dynamic range for a given signal and can be independently tuned for each channel.

**Main results.:**

Low noise component selection yields a no-signal noise floor of just 5.35 ± 0.063. NeuroDAC’s frequency response is characterized with a high pass −3 dB rolloff at 0.57 Hz, and is capable of accurately reproducing a wide assortment of biosignals ranging from EMG, EEG, and ECG to extracellularly recorded neural activity. We also present an application example using the device to test embedded algorithms on a closed-loop neural modulation device, the Medtronic RC+S.

**Significance.:**

By making the design of NeuroDAC open-source we aim to present an accessible tool for rapidly prototyping new biomedical devices and algorithms than can be easily modified based on individual testing needs.

## 1. Introduction

There is an ever growing need to access, process, and understand the complex electrophysiological signals produced by our bodies. Used as indicators for the function or dysfunction of biological systems, successful interpretation of such signals can be used in adaptive therapies to provide precisely timed, personalized treatments to patients [1–9]. Electrocardiograms (ECGs), tissue impedance measurements, and accelerometer based activity signals are used in implantable cardioverter-defibrillators (ICDs) and pacemakers to deliver life saving counter-shocks and rhythmic electrical stimulation to normalize dysrhythmias [7, 8, 10]. More recently, local field potentials (LFPs) measured using deep brain electrodes and electrocorticograms (ECoG) have been used in adaptive deep brain stimulation (DBS) paradigms to treat epilepsy [2, 3, 11], Parkinson’s disease [11, 12], and Tourette’s syndrome [6, 11]. Ongoing research aims to apply these same adaptive neuromodulation tools towards the treatment of mental disorders such as obsessive compulsive disorder and depression [5, 11].

Closed-loop therapies are enabled by implantable medical devices, for example Medtronic’s Micra™ leadless pacemaker (Medtronic, Minneapolis, MN, USA) [8, 10, 13–16], the Neuropace RNS™ (Responsive Neural Stimulator) [1–3, 6, 17–19], and Medtronic’s Activa PC+S™ and Summit RC+S™ devices [9, 20, 21]. Many of these devices are capable of updating their embedded algorithms *in situ,* allowing researchers to investigate and iterate over new processing techniques without the need for additional surgery [4, 6, 9, 20, 21].

While on-the-fly re-programmability is a powerful capability, it is imperative that patient safety not be compromised for the improved flexibility of a therapeutic or research platform. The Medtronic Activa PC+S™ and Summit RC+S™ were designed with a hardware and software ecosystem to enable computer-in-the-loop development of algorithms on an already-implanted device, within clinician set safety limits [4, 9, 20, 21]. However, the opportunity for using computer-in-the-loop techniques is limited to clinical trials where a device is already being implanted to meet a clinical need. Animal models [9, 20] are effective but are expensive and prohibit early innovation by preventing rapid iteration of new designs. Fully computational methods wherein biological systems and device firmware are modeled together [22, 23] allow for rapid testing with programmatic control over all system parameters but are idealized and depend on the accuracy of the model to predict real-life behavior. Ultimately, subjecting a closed-loop device or algorithm to bench-top testing wherein real-life signals can be presented in a repeatable and controlled manner is an indispensable part of the development life-cycle for any such tool [21, 23].

**Figure 1.**
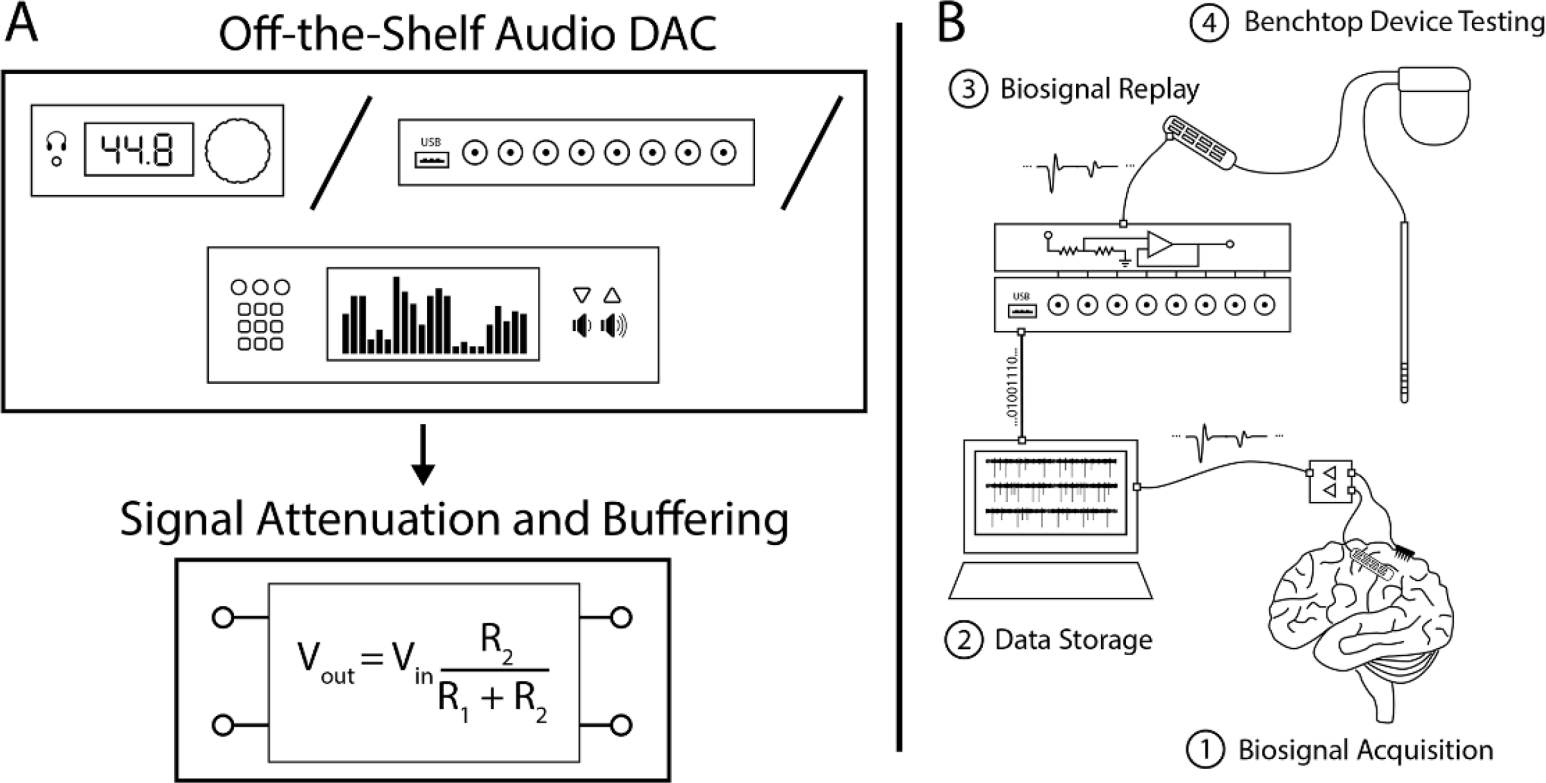
(A) An accessible low-cost consumer grade off-the-shelf audio DAC can be used in conjunction with simple analog attenuation and buffering circuitry to build a biosignal waveform generator. (B) NeuroDAC can be used to play back re-recorded electrophysiology measurements at physiological voltages to perform bench-top verification and validation testing of biomedical devices using real-life signals.

Bench-top signal generation is possible using a variety of tools ranging from laboratory function generators and arbitrary waveform generators to commercially available biosignal specific simulators or ‘patient simulators’ [24–26]. However, general purpose lab equipment are optimized for generic signals with low channel counts and limited ability to reproduce *μ*V scale amplitudes; while commercial simulators are often built for modeling specific electrophysiological events with stereotyped characteristics and are limited to the specific subset of biosignals programmed into the device. Haci *et al* presented a custom built biosignal playback device that uses a RaspberryPi single board computer along with a field programmable gate array (FPGA), audio digital to analog converters (Analog Devices ADAU1966A), and custom circuitry, to provide 32 channels of high quality biosignal playback [27]. However, significant custom circuitry makes it difficult to adapt this design for diverse laboratory needs or to replicate the device without access to circuit schematics, thus limiting its accessibility. Additionally, a custom Raspberry Pi application and FPGA bitstream require significant user know-how to make the device operational. Finally, a microSD card is used to store the playback data meaning that data files exceeding the storage size of the memory card cannot be recreated in full and changing or altering the signal requires re-loading card with the new dataset [27]. In this work we present a methodology for using readily available off-the-shelf audio hardware with a simple analog conditioning circuit to inexpensively recreate a wide range of biosignals for plug- and-play bench-top testing of biomedical devices and provide an open-source design for doing the same (figure 1). Table 1 outlines several currently available options for biosignal generation and their relevant specifications.

## 2. NeuroDAC design

**Figure 2.**
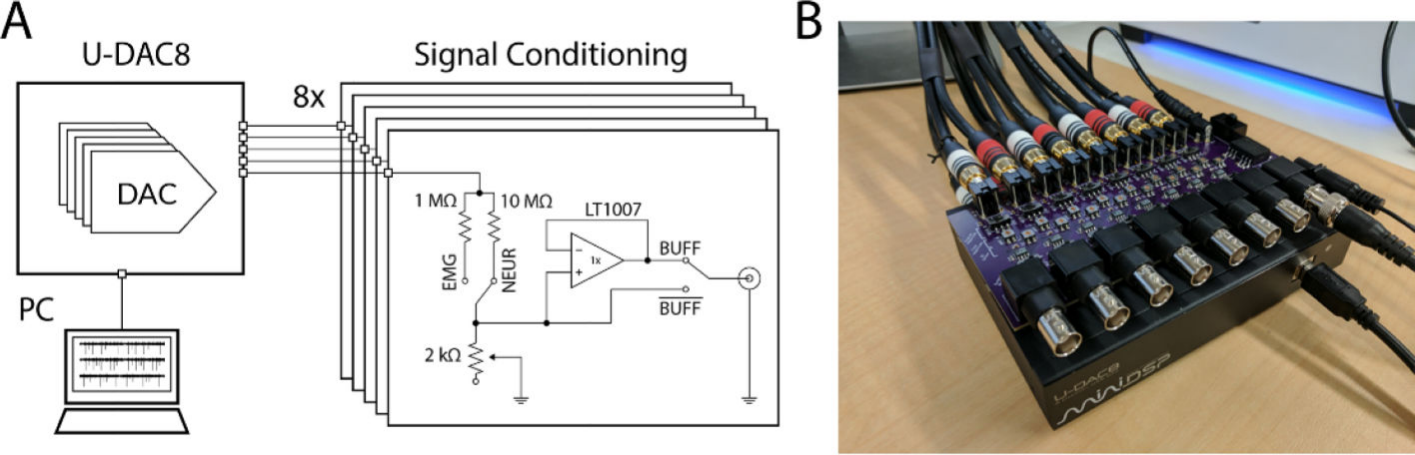
(A) Schematic diagram of a single channel of the NeuroDAC device. A computer is used to stream data to the U-DAC8 via USB and a conditioning circuit converts the line-level voltage output to the small scale signals expected by bioelectronic devices. The conditioning circuit comprises 8 identical channels each containing an adjustable voltage divider circuit and a low noise op-amp based buffer with switches to independently configure the circuit as desired by the user. (B) An image of the NeuroDAC device depicting the U-DAC8 (miniDSP, Hong Kong) audio DAC and the custom-built attenuation circuit with BNC outputs for easily connecting to downstream devices. The U-DAC8 is powered through the conditioning board so only one power adapter is required.

Biosignals comprise spectral information from less than 1 Hz to 10 kHz at voltage amplitudes from less than 1 *μ*V to 10’s of mV [33–36]. A biosignal waveform generator must be capable of reproducing signals in this voltage and frequency range with good signal to noise properties, limited signal distortion, and with high accuracy. Humans are capable of perceiving auditory stimuli from 20 Hz to 20 kHz [37], a range spanning a large proportion of common biosignals. High fidelity consumer audio equipment is optimized for faithfully reproducing waveforms in this frequency domain and often feature frequency responses with high pass cutoff frequencies lower than 20 Hz, making them useful for low frequency signals as well. In this work, we leverage a low-cost high quality off-the-shelf USB enabled audio digital to analog converter (DAC), the U-DAC8 (miniDSP, Hong Kong), to convert digitally stored biosignal waveforms into the analog domain. Compatibility with standard USB audio drivers results in ‘plug-and-play’ communication with the device from any modern computer. A custom designed, simple, printed circuit board (PCB) is used for further signal conditioning and can be easily modified to suit the needs of different users (figure 2). We’ve termed this device ‘NeuroDAC’.

The U-DAC8 audio DAC is based on the AK4440 chipset (Asahi Kasei Microdevices, Tokyo, JP) with 8, 24 bit, independent single ended output channels and a total harmonic distortion of 0.006% [38]. A user defined sample rate can be set from 44.1 kSPS to 192 kSPS. Built-in low pass filtering results in a DC coupled passband with a cutoff frequency between 8.1 kHz and 87 kHz depending on the sample rate and filter characteristics chosen by the user [38]. Taking advantage of these capabilities, the NeuroDAC device can play up to 8 channels of full bandwidth biosignals simultaneously. It should be noted that while the filter characteristics of the AK4440 chipset are ideal for biosignal playback, the U-DAC8 device implements its own hardware filtering that results in a load dependent highpass cuttoff frequency of 1 Hz with a 47 kΩ load. Conveniently, the U-DAC8 is supported by standard ASIO and ALSA drivers and is recognized as a USB Audio Class 2.0 device by the host computer [38]. Therefore, any application capable of streaming audio to a USB audio device can send data to NeuroDAC and custom applications can be implemented using common programming languages such as MATLAB (The Mathworks, Inc., Natick, MA, USA) and Python.

The AK4440 and thus the U-DAC8 are meant to drive audio devices at line-level voltages with a stated typical maximum output voltage of 2.12 V_rms_, measured at 6.4 V_pk–pk_ or 2.26 V_rms_ (data not shown). These voltages are far higher than those recorded in electrophysiological measurements. Therefore, the output signal of the U-DAC8 must be lowered to achieve proper amplitudes for biosignal playback. Rather than digitally scale the DAC’s input from the PC by reducing the audio device driver’s playback volume, which would dramatically reduce the dynamic range of the system and potentially subject the output to the quantization noise of the DAC, analog voltage division was achieved using a custom designed PCB (figure 2(A)). NeuroDAC must be able to support playback of a wide range of biosignals including *μ*V level signals such as low amplitude intracortical extracellular microelectrode recordings or EEG [33, 36] to mV level signals such as ECG [35]. To maximize the dynamic range of the system, voltage division can be adjusted using two different controls. A switch allows coarse selection between a 1 MΩ resistor and a 10 MΩ resistor. Paired with a 2 kΩ potentiometer, the circuit allows for fine tuned control of the voltage divider ratio from 501:1 to 100 001:1 (see schematic in figure 2(A)). As a result, the output dynamic range of NeuroDAC can be continuously tuned from 12.78 mV_pk–pk_ to 64 *μ*V_pk–pk_. These values were calculated using the measured 6.4 V_pk–pk_ maximum output of the U-DAC8 and by assuming the potentiometer is kept above a minimum resistance of 100 Ω. At a given analog setting, smaller amplitudes can also be achieved by digitally scaling the values streamed to the DAC, with a proportional loss in dynamic range. Since all modifications to the original audio DAC are confined to the analog domain and occur downstream of the digital conversion, NeuroDAC’s resolution matches that of the U-DAC8. If calibrated appropriately, the entire dynamic range of the 24 bit DAC can be used for signals within these ranges. Importantly, the conditioning circuitry is replicated independently for each of the 8 channels, therefore settings can be tailored separately for each signal being played from the device.

While many uses for NeuroDAC involve playing a signal into the well defined high impedance input of a recording amplifier, it is also possible that the device will be used to play signals into an unknown impedance, for example a saline bath to emulate *in vivo* conditions. Output buffers were implemented to ensure signal integrity regardless of the load impedance presented to the device. However, the addition of active circuitry leads to the possibility of introducing electrical noise into the output signal. Therefore, the buffer circuit was carefully designed to minimize additional noise. A switch is also provided to remove the buffer from the signal chain if an unbuffered output is preferred.

**Figure 3.**
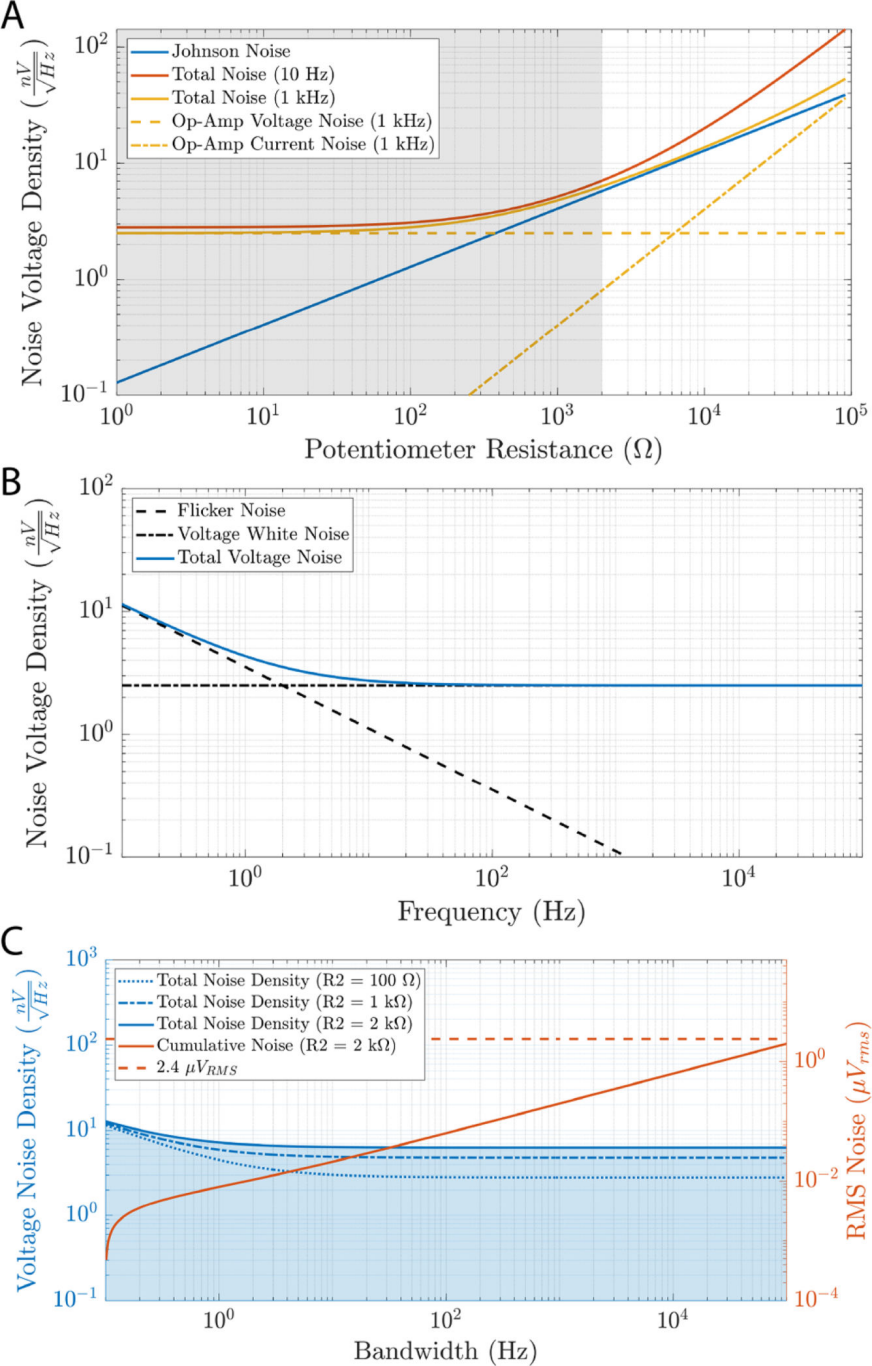
(A) A low voltage noise op-amp was chosen as the output buffer because this type of noise dominates in the operating regime of the circuit. A comparison is given between the voltage and current noise of the LT1007 op-amp and the Johnson noise of the voltage divider circuit for different settings of the potentiometer (switched resistor at 1 MΩ) at 1 kHz. The shaded region represents the range of resistances available for the 2 kΩ potentiometer used in NeuroDAC. Theoretical total noise accounting for all of these sources is also shown at 10 Hz and 1 kHz. (B) The full theoretical voltage noise spectrum of the LT1007 op-amp including low frequency flicker noise. (C) The total estimated noise spectra of the NeuroDAC analog circuitry at potentiometer settings of 100 Ω, 1 kΩ, and 2 kΩ. Cumulative RMS noise is indicated in blue as the bandwidth of the system is selected from 0.1 Hz to the value indicated on the horizontal axis. The shaded blue region indicates the integral area underneath the noise density curve for the worst-case 2 kΩ potentiometer setting. A dashed line indicates the reported noise floor of the Intan RHD2132 biosignal amplifier for reference (2.4 *μ*V_rms_ [41]).

There are several potential noise sources to contend with when handling *μ*V level waveforms; figure 3 depicts some of these. Johnson noise is thermal noise inherent to any real resistance in a circuit. Its value is given by the equation:

$$e_{\text{Johnsoo}}=\sqrt{4kTR},$$

with units $\frac{V}{\sqrt{Hz}}$ of white noise spectral density. Where *k* is Boltzman’s constant, *T* is the temperature of the device, and *R* is the resistance [39]. Johnson noise in the NeuroDAC output circuit is determined by the parallel value of the voltage divider resistors and is therefore dominated by the 2 kΩ potentiometer and changes based on the selected position. The LT1007 operational amplifier (Analog Devices, Norwood, MA, USA) was chosen for the output buffer due to its low input referred voltage noise specification of $2.5\;\frac{\text{nV}}{\sqrt{\text{H}z}}$ at 1 kHz (typical). To achieve low voltage noise, the LT1007 device compromises with its input referred current noise specification $\left(0.4\;\frac{\text{pA}}{\sqrt{\text{Hz}}}\right)$ [40]. Current noise is converted to additional voltage noise through the source impedance seen by the op-amp and therefore also varies with potentiometer setting. Figure 3(A) depicts the contributions of each of the noise sources discussed, as well as the theoretical total noise at both 10 Hz and 1 kHz for different potentiometer settings. The shaded gray area shows the operational region of the potentiometer used in NeuroDAC (0–2 kΩ). In this regime the contribution of current noise is small compared to voltage noise, justifying the selection of an op-amp prioritizing its voltage noise characteristics.

At low frequencies, flicker noise (also called 1/*f* noise) is present. The LT1007 has a typical 1/*f* corner frequency of 2 Hz [40]. Figure 3(B) depicts the relative contributions of flicker noise and voltage noise in the 0.1 Hz–100 kHz band as well as the resulting theoretical noise floor contributed by the LT1007 to the output of the NeuroDAC. Figure 3(C) shows the total theoretical noise density of the output conditioning circuit for different potentiometer settings. Cumulative RMS voltage noise is shown for a ‘worst case’ potentiometer value of 2 kΩ given as the integral the noise density over a bandwidth from 0.1 Hz to the frequency indicated on the independent axis. For comparison, the theoretical noise floor of the Intan RHD2132 electrophysiology amplifier (2.4 *μ*V_rms_ [41]) is overlayed, representing a commercially available device used for measuring small biosignals. It can be seen that the noise added by NeuroDAC remains lower than the noise floor of a typical biosignal amplifier even for full bandwidth recordings from 0.1 Hz to 10 kHz.

## 3. NeuroDAC validation

Bench-top validation of the NeuroDAC system was performed to characterize the device’s noise properties, frequency response, and to demonstrate its ability to recreate digitally stored real-world biosignals. All validation testing was performed using the OpenEphys data acquisition system [42] along with an Intan RHD2132 amplifier headstage (Intan Technologies, Los Angeles, CA, USA) to record the output of the NeuroDAC since standard electrical measurement equipment is not designed for measuring *μ*V signals.

### 3.1. Noise characteristics

**Figure 4.**
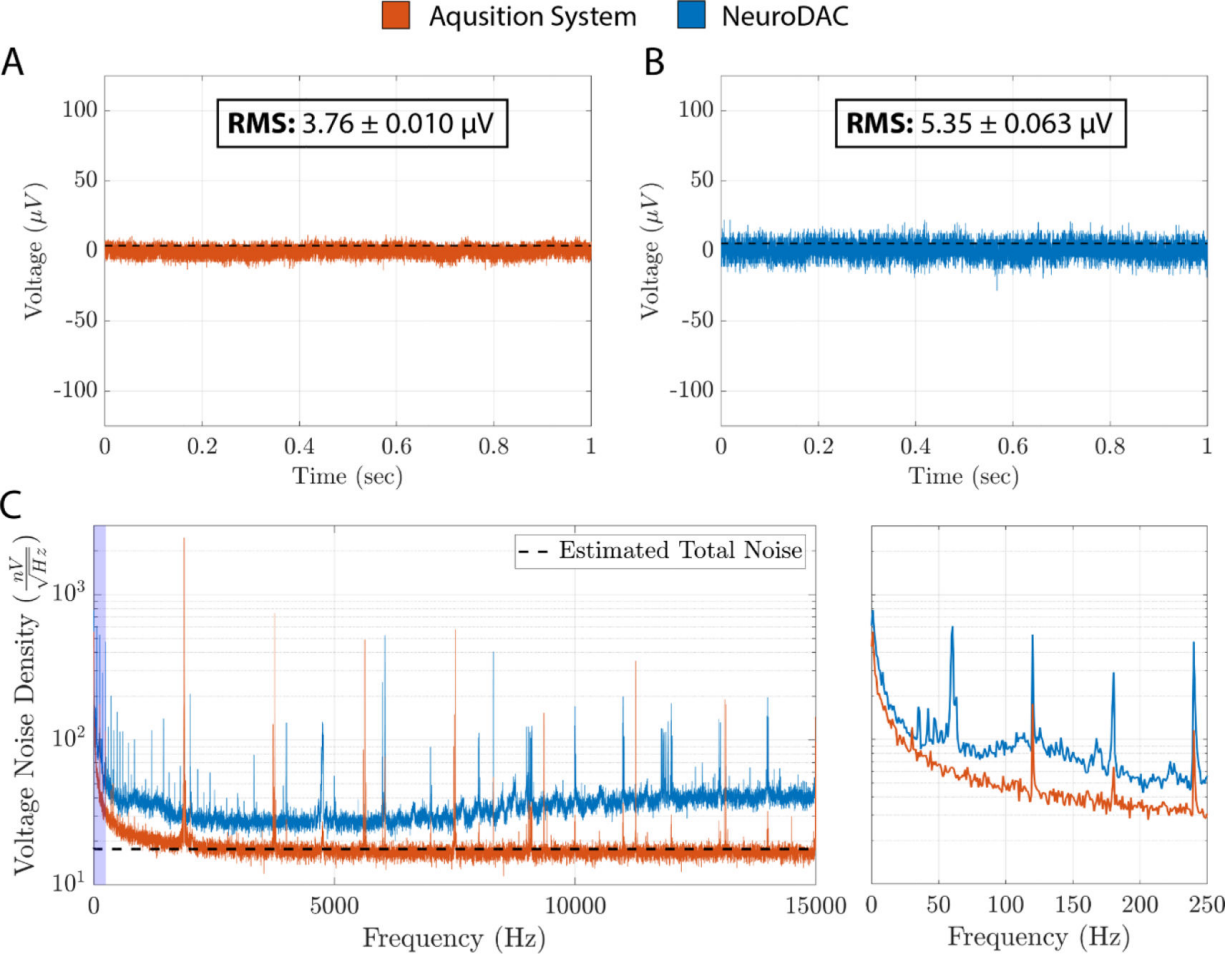
(A) A raw 1 second example trace of the Intan RHD2132 amplifier and OpenEphys data acquisition system noise floor [42]. Mean RMS noise floor (± std.) for all 5, 10 second long, recordings is overlayed as a dotted black line. (B) A raw 1 second example trace of the NeuroDAC noise floor recorded through the Intan/OpenEphys acquisition system. Mean RMS noise floor (± std.) for all 5, 10 second long, recordings is overlayed as a dotted black line. (C) A comparison of the estimated noise floor density between the data acquisition system with and without the NeuroDAC attached. The theoretical estimation of the noise density is given as a black dotted line. An expanded view of the frequency range from 0 to 250 Hz (blue highlighted region) is shown on the right.

System noise was assessed by connecting one NeuroDAC channel to an input of the Intan headstage while the NeuroDAC was powered on but not actively streaming. A separate Intan channel was shorted to its reference to serve as a control measurement of the recording system’s noise floor. On-board toggles for the NeuroDAC channel under test were configured with the channel’s coarse amplitude adjustment switch set to 10 MΩ and the potentiometer set approximately at it is nominal value of 1 kΩ. Output buffering was enabled on the selected channel while all other channels had their buffers disconnected. The outputs of 6 of the remaining 7 channels were 50 Ω terminated, while the final channel’s output connector shell was used as a grounding point. A Faraday cage, referenced to the OpenEphys acquisition system ground, was implemented to minimize the influence of external noise sources. In some cases, it was empirically determined that additionally connecting the Faraday cage to the NeuroDAC’s reference through an AC coupling capacitor helped further reduce noise. The Intan amplifier’s integrated filters were set to the maximum bandwidth of 0.09 Hz–20.5 kHz. Figure 4 illustrates the measured noise characteristics.

To generate these results, 5 sequential 10 second long recordings were captured using the configuration described above. A 1-second long snippet of raw data was extracted from one of the recordings for both the grounded Intan channel (figure 4(A)) and the NeuroDAC connected channel (figure 4(B)). The NeuroDAC channel contained a noticeable but small increase in noise compared to the base noise floor of the OpenEphys acquisition system. RMS voltage was calculated independently for each of the 5 recordings and averaged resulting in a mean RMS noise floor of 3.76 ± 0.01 *μ*V_rms_ (standard deviation) for the grounded amplifier channel and 5.35 ± 0.06 *μ*V_rms_ (standard deviation) for the NeuroDAC channel, an increase of just 1.59 *μ*V_rms_. These mean RMS values are indicated as a dotted line overlays in figure 4(A) and B. To further quantify system noise, each of the 5 recordings for both channels were concatenated and the noise spectral density was estimated using Bartlett’s method in which the average periodogram was computed for 50 non-overlapping windows of the timeseries data (each 10 second recording split into 1 second epochs). Figure 4(C) shows the resultant noise spectra along with an overlay of the estimated voltage noise density based on the theoretical noise calculations described above and the published noise floor of the Intan amplifier (2.4 *μ*V_rms_) [41]. Excess noise observed in the NeuroDAC may have been caused by digital switching noise or other environmental factors that were not accounted for in the theoretical models above, possibly due to long BNC cabling that could not be fully concealed within the Faraday cage. Careful consideration and optimization of the recording environment should be performed for all electrophysiologial experiments. Despite this small discrepancy, the NeuroDAC presents a low noise floor of just 5.35 *μ*V_rms_.

### 3.2. Output linearity

**Figure 5.**
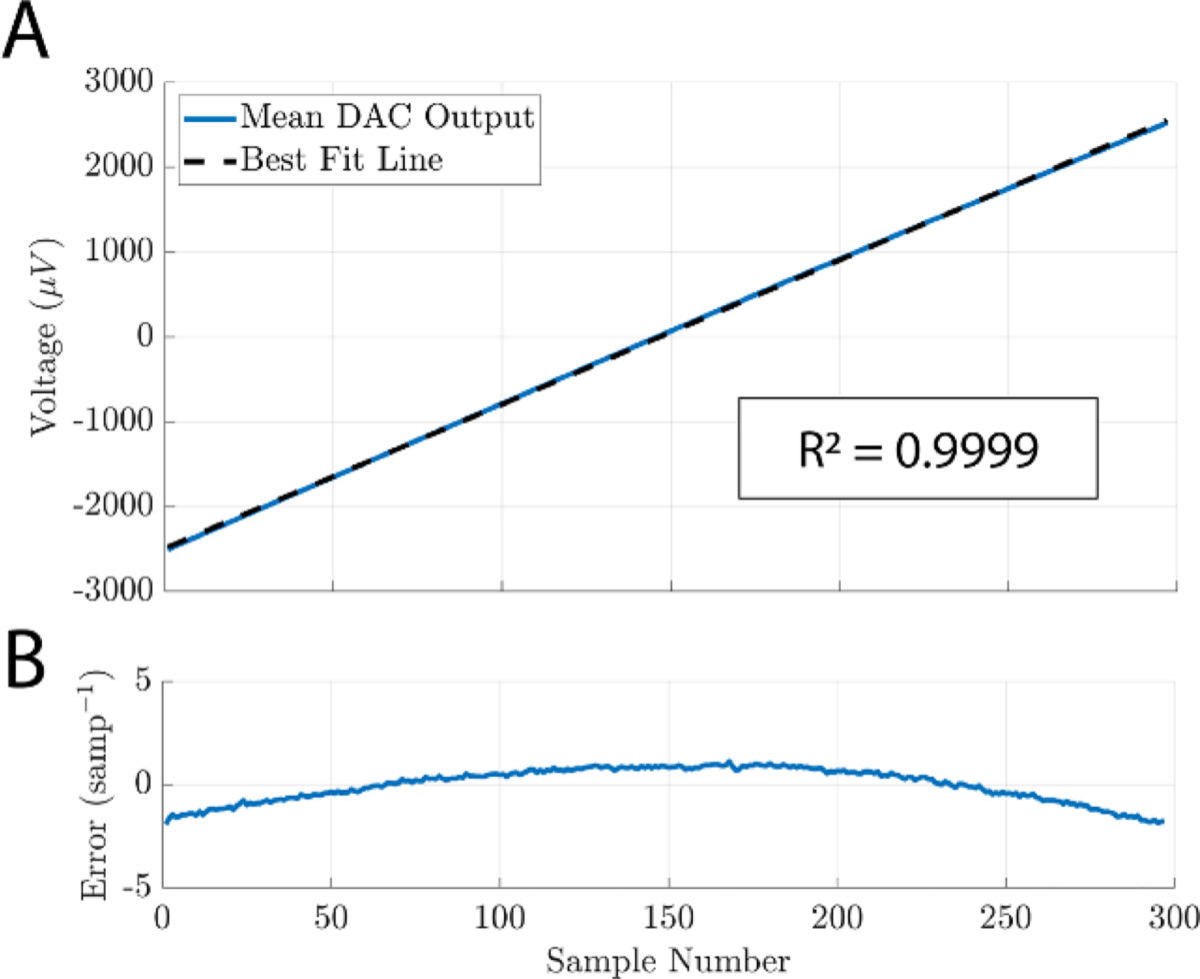
(A) Mean recorded DAC output for 10 repetitions of a linear, full-scale, sweep of input values. Black dotted line is the best-fit-line of the result found using least squares linear regression (*R*^2^ value reported in boxed annotation). (B) Per sample residuals for the waveform shown in (A). Values normalized to the slope of the best-fit-line.

Selecting a particular setting for the the coarse and fine adjustments of NeuroDAC’s attenuation circuit results in selecting the theoretical maximum and minimum voltage that can be produced by the system. Output linearity was measured to verify that a given digital input within the 24-bit full scale range of the DAC yields the expected analog output from within the chosen extrema. A linearly increasing set of digital inputs, generating a ramp signal, spanned the full range of possible values and was played into the NeuroDAC. Ten ramps were repeated in succession. Each ramp lasted 0.01 s to minimize the effects of the non-zero high pass frequency response described in the following section, and was followed by a 1 s buffer of 0’s before the next ramp began. The attenuation circuit was configured to yield a −2.5 mV to +2.5 mV output dynamic range by setting the coarse switch to 1 MΩ and the potentiometer to just below its 1 kΩ nominal value. This range represents a wide sweep of output voltages while staying well within the specified maximum input range of the Intan amplifier used to measure the signals (±5 mV, [41]). All ten recorded ramp signals were extracted and averaged to minimize the effects of white noise. A linear regression was performed to identify the best-fit-line (figure 5(A)) and the residuals were normalized to the slope of the best-fit-line providing a figure-of-merit indicating how far the output strayed from an ideal line relative to the expected increase between two adjacent samples (figure 5(B)). The result shows good linearity (*R*^2^ = 0.9999) deviating from −1.91 sample^−1^ to +1.15 sample^−1^.

### 3.3. Frequency response

In order to faithfully reproduce the widest array of biosignals possible, it is important that NeuroDAC maintain minimal distortion over it is operating regime. To assess its capabilities, the system’s frequency response was characterized. 40 individual sine waves were generated, logarithmically spaced spanning the frequency range of 0.1 Hz to 10 kHz. Each sine wave was played for a duration equivalent to 5 periods of oscillation, thus lower frequencies were played for a longer duration than high frequencies. Each waveform was preceded by a short synchronization pulse to aide with estimating phase shift. A blanking time of 2 s followed each sync pulse, as well as following each sine wave (2 periods in duration) to allow for any transients to settle between successive waveforms. The NeuroDAC played back the signals at its full sample rate of 192 kHz in order to best recreate the highest frequencies. The Intan amplifier was again set to use its widest bandwidth of 0.09 Hz–20.5 kHz so that its influence on the signals was minimized. To quantify the magnitude and phase of the recorded test signals, a sinusoid of equal frequency was fit to the data and its amplitude and phase estimated using non-linear least squares estimation in MATLAB (The Mathworks, Inc., Natick, MA, USA). Only fits with a *R*^2^ goodness of fit metric above 0.95 were used in the analysis (eliminating the highest frequency tested, 10 kHz, due to a low number of samples/period).

**Figure 6.**
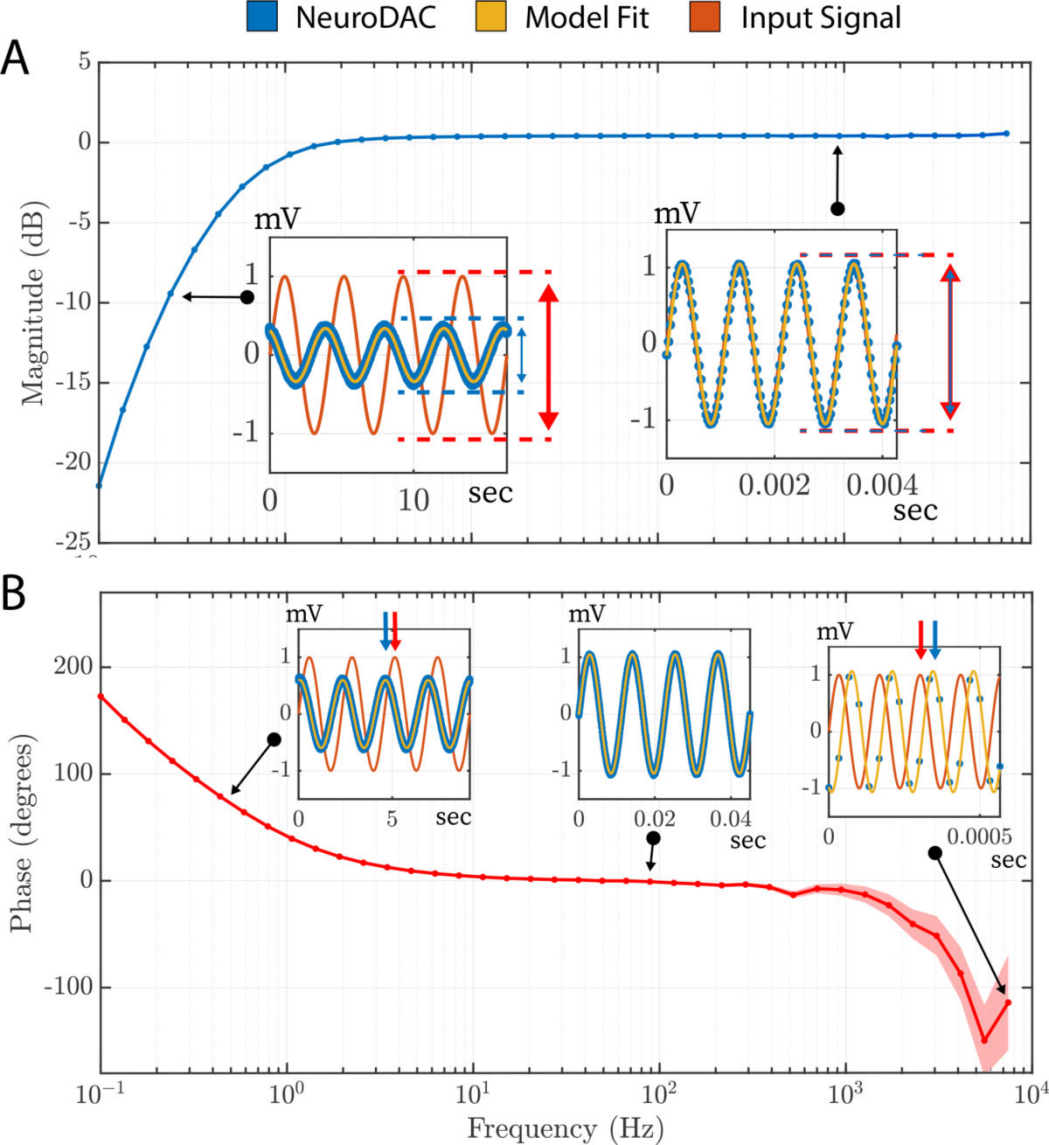
(A) Magnitude of the frequency response of the NeuroDAC system. Amplitude is attenuated for signal components lower than 1 Hz, but are accurately represented up to 10 kHz. Sub-figures call out individual data points used in the estimation, showing the original signal, raw playback signal, and model fit used to estimate amplitude parameter. (B) Phase of the frequency response of the NeuroDAC system. A phase shift is observed at the extrema of the operating bandwidth, however prediction accuracy may be decreased at higher frequencies due to the low sample/period ratio of the 30 kSPS data acquisition system. Sub-figure callouts indicate individual data points used to calculate phase shift.

Figure 6(A) shows the magnitude of the frequency response indicating how the amplitude of different frequency components were altered by the system. A nominal amplitude of 1 mV was chosen for this test which was large enough to give good signal-to-noise headroom for accurately measuring the diminishing amplitudes within the frequency response roll-off region. For most of the frequency range, the amplitude was well preserved (0 dB attenuation). There was a high pass −3 dB cutoff frequency of 0.57 Hz (identified through linear interpolation) consistent with the value quoted by the U-DAC8 manufacturer of 1 Hz at 47 kΩ load. A −20 dB/decade rolloff was observed indicating a first order filter profile. Phase shift was also visible at the lowest and highest frequencies. It should be noted that due to the 30 kSPS sample rate of the Intan’s ADCs, higher frequency signals were necessarily more sparsely sampled resulting in higher uncertainty in these values. To indicate this, the shaded region in the phase plot depicts the estimated phase ± the phase shift in degrees due to a 1 sample shift in alignment. The region of uncertainty grows as one ADC sample becomes a larger portion of the signal’s period. As indicated, most of the spectral components of common biosignals are preserved during NeuroDAC playback. However, the altered signal characteristics at high or low frequencies are important to understand so the user can be informed about how the system will handle specific features of their data.

### 3.4. Playback in saline

It may not always be possible or desirable to play signals from NeuroDAC directly into the high impedance input amplifiers of a biomedical device. For instance, when testing the electrical properties of a novel electrode design or when attempting to interface with probes that are too small or delicate to connect to directly, saline may be used as a tissue substitute with similar electrochemical properties. In such cases, NeuroDAC may be used to replay signals into the saline near the electrodes being tested resulting in electric field fluctuations similar to those encountered *in vivo.* However, the electrode-electrolyte interface of the electrodes used to inject NeuroDAC’s signal into the saline, as well as the conductive properties of the saline itself, would result in an uncertain and likely non-ideal impedance across NeuroDAC’s output terminals. Buffers were added to NeuroDAC’s conditioning circuit to prevent any undesirable loading effects caused by playing a signal into an unknown downstream impedance.

**Figure 7.**
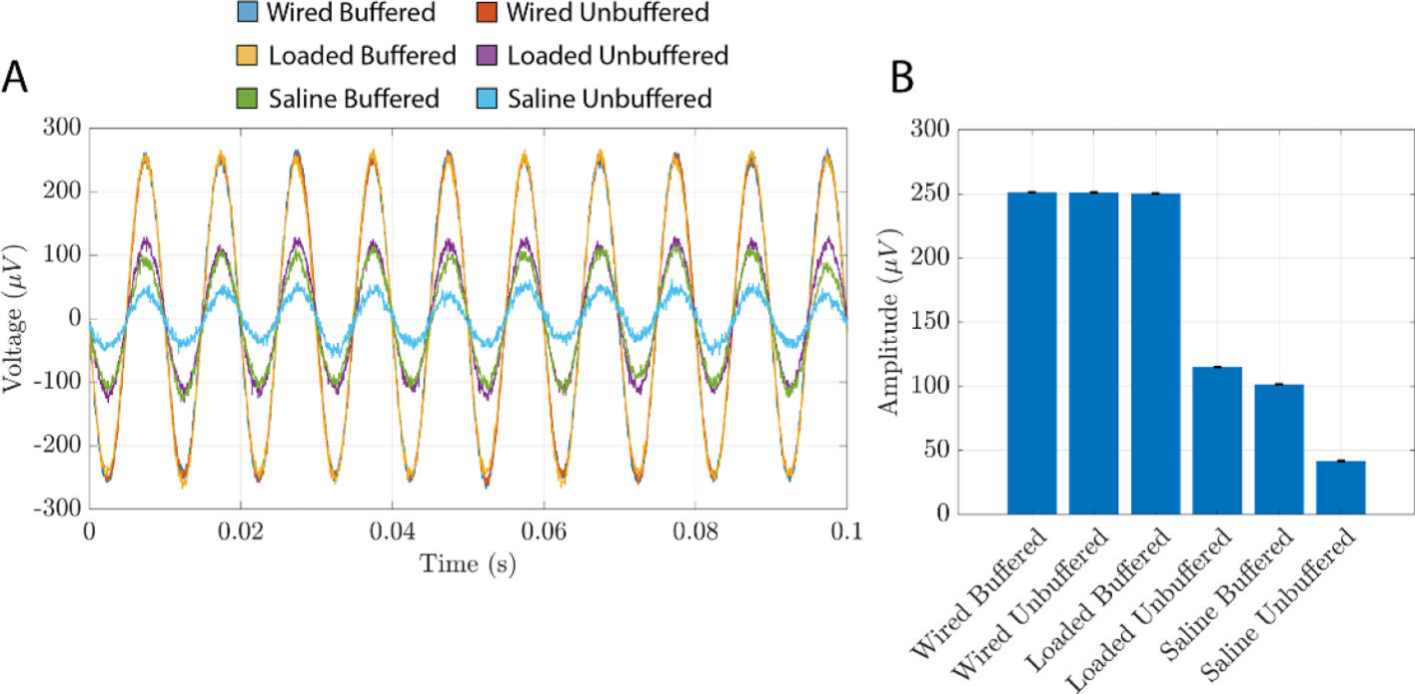
Measured output voltage of NeuroDAC while playing a 100 Hz, 250 *μ*V sine wave into saline. The output was measured with and without buffering outside of the saline (‘Wired’), loaded by the saline (‘Loaded’), and through the saline using a tungsten electrode (‘Saline’). (A) A 0.1 s window of the raw recorded data from each condition. The waveforms were phase aligned in post processing to allow for easy comparison of amplitudes. (B) The estimated amplitude of the recorded waveform in each condition; calculated from 10 s of recording. Error bars indicate the 95% confidence interval of the estimated amplitude.

To ensure that the buffers performed as intended, NeuroDAC was used to play a 100 Hz, 250 *μ*V, sine wave into a 0.9% saline solution and measured by the Intan amplifier. Figure 7 depicts the results. Three electrode configurations were compared. In each configuration, the 100 Hz signal was played twice: once with the DAC’s output buffers enabled, and once with them disabled. First, as a control, the Intan’s input and reference were directly connected to the DAC’s output terminals without saline (labeled ‘Wired Buffered’ and ‘Wired Unbuffered’). The measured waveform was the same in both buffered and unbuffered cases (251.37 and 251.25 *μ*V respectively) indicating that the high input impedance of the biosignal amplifier alone yielded no observable loading effects. Next, leaving the amplifier directly connected to the NeuroDAC’s output, two surface EMG electrodes were also connected to the output terminals, using standard laboratory wire clips, and submerged in the saline solution (labeled ‘Loaded Buffered’ and ‘Loaded Unbuffered’). With the buffer connected, the measured amplitude was 250.44 *μ*V. However, with the buffer unconnected, the signal’s amplitude dropped to 115.01 *μ*V. Finally, a tungsten wire electrode was connected to the amplifier’s input while its reference remained connected to the NeuroDAC’s reference (both were connected to the same EMG electrode). The tip of the tungsten wire was lowered into the saline solution and another set of measurements taken; yielding measured amplitudes of 101.47 *μ*V in the buffered and 41.72 *μV* in the unbuffered conditions (labeled ‘Saline Buffered’ and ‘Saline Unbuffered’ respectively). The tungsten electrode tip was approximately 1 cm from the surface of the signal driving EMG electrode and the two EMG electrodes were separated by approximately 4 cm. A 150 ml beaker was used to contain the saline during the experiment. All reported amplitudes were estimated by fitting a 100 Hz sine wave to 10 s of recorded signal using non-linear least squares estimation in MATLAB (The Mathworks, Inc., Natick, MA, USA).

These tests indicate that the buffer helps to maintain the desired DAC output voltage even while being loaded by the electrochemical cell’s impedance. Measured from within the saline bath, the buffered voltage amplitude was reduced compared to the DAC’s actual output voltage. This reduction is to be expected as the tungsten electrode was measuring the electric field some distance away from the DAC’s output electrode and the electrode-saline interface impedance of the acquisition system must also be considered. However, the buffer’s effect in saline is to maintain a higher measured amplitude than with no buffer present. It is therefore recommended that the ‘buffer’ switch be selected when using NeuroDAC in situations where downstream impedances are unknown or would cause loading effects when combined with its 2 kΩ, unbuffered, output impedance.

### 3.5. Biosignal playback

**Figure 8.**
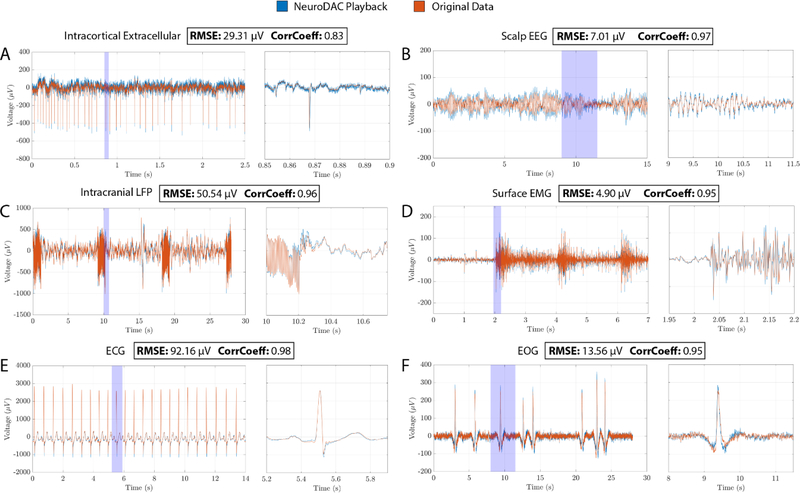
A set of 6 different calibrated biosignals played through the NeuroDAC, overlayed and aligned to the original signal. In each sub-figure, a subset of the 30 second recording is shown on the left and an expanded view of the highlighted blue region is shown on the right highlighting a feature of interest for each signal. Different time and voltage scales are used due to the heterogeneity of signal characteristics. The signals represented are: (A) a microelectrode array recording of action potentials from a nonhuman primate; a single unit action potential is highlighted, (B) scalp-EEG recorded from a human subject; an alpha oscillation is highlighted, (C) intraoperative intracranial recording from a human subject; 130 Hz stimulation artifact is highlighted, (D) surface EMG activity recorded from an awake sheep; evoked activity from spinal cord stimulation is highlighted, (E) ECG recorded from a human subject; a single waveform is highlighted, and (F) EOG activity recorded from a human subject; a single blink event is highlighted. For more information about each signal, please refer to the main text body.

As a demonstration of the NeuroDAC’s capabilities, 6 different biosignals were replayed through the device and compared to their original digital recording (figure 8). In all cases, the digital signals were first run through a third order, zero-phase, digital Butterworth filter to eliminate any DC offset in the recording and to avoid distortion caused by the high pass frequency response of the NeuroDAC. Before playback, each signal was re-sampled from its original sample rate up to the U-DAC8’s highest 192 kSPS to achieve the best digital resolution. To calibrate the NeuroDAC, the signal’s most positive or negative value was identified and a custom script determined the appropriate voltage divider settings such that the maximum output amplitude of the DAC matched this signal extremum. A 10 Hz sine wave was then played at a known amplitude and the circuit’s potentiometer was manually adjusted to match the output voltage to the expected value.

By mapping the signal’s maximum to the output amplitude of the DAC, the best possible dynamic range was achieved for each signal. In each case, a 30 second dataset was streamed from a host computer to the NeuroDAC and the signal was re-recorded and digitized at 30 kSPS using the OpenEphys acquisition system [42] and Intan RHD2132 amplifier headstage (Intan Technologies, Los Angeles, CA, USA). A built in 0.09–7500 Hz filter was applied to all signals by the Intan amplifier, except for the extracellular microelectrode array recording which was filtered from 0.09 Hz–15.34 kHz due to its higher frequency content. After acquisition, the recordings were down-sampled to match the original sample rate for each dataset. Cross-correlation was used to align the re-recorded waveforms with the input signal. Correlation coefficients (ranging from 0.83 to 0.98) and root mean square error (RMSE—ranging from 4.90 to 92.16 *μ*V) metrics are given for each dataset indicating that the waveforms replayed from the NeuroDAC closely match the original data (figure 8). It is noteworthy that RMSE values are susceptible to the calibration of the analog circuitry. Calibration must be done carefully to achieve the best results. Here we give a brief description of each signal used.

#### 3.5.1. Extracellular recording of action potentials from primary motor cortex of a nonhuman primate

*Data presented were collected in accordance with the Brown University Institutional Animal Care and Use Committee (IACUC) under the approved protocol 1807000360*. Large amplitude, single unit action potentials are shown. The original acquisition was captured at 20 kSPS and up-sampled to 30 kSPS using a Utah style, silicon microelectrode array and a CerePlexW™ head-mounted wireless neural amplifier and RF transmitter (Blackrock Microsystems, Salt Lake City, UT, USA). Since action potentials can contain frequency content up to 7 kHz [34], the integrated bandpass filter on the Intan headstage was configured to 0.09 Hz–15.3 kHz. No additional filtering was performed aside from the high pass filter applied to all 6 signals described above (figure 8(A)).

#### 3.5.2. Scalp-EEG activity recorded from electrodes placed over occipital cortex of an awake human subject at rest with eyes open or closed

*EEG data presented were collected in accordance with the recommendations of the federal human subjects regulations and under protocol FWA 00004460 approved by the Brown University Institutional Review Board*. Characteristic alpha oscillations (8–12 Hz) are clearly shown during periods when the participant’s eyes were closed. Activity was referenced to the right mastoid and captured at 25 kSPS using the Brain Vision ActiCHamp Plus EEG acquisition system (Brain Vision LLC, Morrisville, NC, USA). Data were demeaned and re-referenced to the common average across recording electrodes, bandpass filtered using a 1–100 Hz Butterworth filter, and decimated to 1 kSPS (figure 8(B)).

#### 3.5.3. Intraoperative intracranial recording during electrical stimulation of the left ventral capsule/ventral striatum of a human subject

*Intraoperative intracranial data presented were collected in accordance with the recommendations of the federal human subjects regulations and under protocol H-43036 and H-40255 approved by the Baylor College of Medicine Institutional Review Board*. Periodic bursts of stimulation artifact are shown. Electrical stimulation was delivered at 130 Hz in 1 second on, 8 second off epochs. Signals were recorded at 2 kSPS using the NeuroPort™ neural signal processor (Blackrock Microsystems, Salt Lake City, UT, USA), demeaned, bandpass filtered from 0.5 to 250 Hz, notch filtered at 60, 120, and 180 Hz, and common average re-referenced to all other contacts on the same lead. NeuroDAC can be used to reliably reproduce complex signal features such as stimulation artifact to aide in, for example, the development of artifact rejection tools and techniques (figure 8(C)).

#### 3.5.4. Differential surface EMG activity recorded from the right peroneus longus muscle of an awake sheep during epidural spinal cord stimulation

*EMG data presented were collected in accordance with the Brown University Institutional Animal Care and Use Committee (IACUC) under the approved protocol 19–04-0002*. Stimulation was delivered in 2 second intervals and the evoked muscle activity is clearly visible. EMG was recorded at 1.26 kSPS and 20–450 Hz bandpass filtered (2-pole high pass, 4-pole low pass) using the Delsys Trigno Avanti™ wireless sensor system (Delsys, Natick, MA, USA). Spinal cord stimulation was performed using a custom designed epidural spinal cord electrode array (Micro-Leads Medical, Somerville, MA, USA) and a Grapevine Nomad™ external simulator (Ripple Neuro, Salt Lake City, UT, USA) (figure 8(D)).

#### 3.5.5. ECG recorded from a human subject at rest

*ECG data presented were collected in accordance with the recommendations of the federal human subjects regulations and under protocol H-40255 approved by the Baylor College of Medicine Institutional Review Board*. Individual heartbeats are depicted expressing the canonical P-wave, QRS-complex, and T-wave waveshapes. The recording electrode was placed directly underneath the left pectoralis major muscle and referenced to the left mastoid. Data was acquired at 30 kSPS using an OpenEphys data acquisition system [42] and an Intan RHD2132 single ended amplifier (Intan Technologies, Los Angeles, CA, USA). After acquisition, the signal was decimated to 1 kSPS, low pass filtered (FIR, 94th order, 100 Hz passband, 130 Hz stopband) and notch filtered (FIR, 100th order, 60 Hz) (figure 8(E)).

#### 3.5.6. Vertical eye movements recorded using EOG electrodes placed above and below the eye of a human subject during spontaneous blinking

*EOG data presented were collected in accordance with the recommendations of the federal human subjects regulations and under protocol FWA 00004460 approved by the Brown University Institutional Review Board*. Individual eye blink events are highlighted and clearly visible as large deflections from baseline activity. Data were acquired at 25 kSPS using an auxiliary analog input channel of the Brain Vision ActiCHamp Plus EEG acquisition system (Brain Vision LLC, Morrisville, NC, USA). The signal was then demeaned, filtered using a 1–100 Hz Butterworth bandpass filter, and decimated to 1 kSPS (figure 8(F)).

## 4. Using NeuroDAC

The MiniDSP U-DAC8 was chosen for this work because of its low cost, use of high quality audio-grade components, wide signal bandwidth, and out-of-the-box USB support. However, the advantage of designing around standard audio equipment is that any accessible audio DAC could be used in conjunction with the signal conditioning circuit to yield similar functionality (Table 2). Thus, the NeuroDAC paradigm is generally applicable to an extensive set of readily available off-the-shelf products; making it trivial to adapt the design to countless combinations of features, specifications, and software workflows depending on user needs. Here we will describe one such workflow using the U-DAC8.

To a host machine, the U-DAC8 appears as a standard 8 channel, USB Audio Class 2.0 device. ASIO and ALSA compatible drivers are available from the manufacturer, providing support for the device on Windows, MacOS (driverless), and Linux operating systems. Therefore, any application capable of streaming audio on a computer can be used to operate NeuroDAC. Our group has achieved programmatic control of the device using the common programming languages MATLAB (using the Audio Toolbox) and Python (using the python-sounddevice package) [43]. Instead of requiring data to be saved to an on-board storage device for playback [27], buffered real-time streaming means there is virtually no data file size limit with NeuroDAC. Buffer size and sample rate can be adjusted via the driver to optimally support pause-free playback on a given host machine.

Output signal amplitude is controlled in the analog domain using an on-board potentiometer to set a voltage division ratio. For this reason, calibration must be performed prior to accurately reproducing biosignal voltages. Several approaches are possible. A channel can be calibrated such that its maximum output voltage is set at a fixed, known, value. The desired signal is normalized to this value to yield the correct amplitude. In this way, a ‘set it and forget it’ strategy is adopted where calibration is only required once, and all future signals are adjusted to match that calibration. However, if playback signals vary significantly in their dynamic range, a fixed calibration may not be appropriate. In this case, channels may be recalibrated prior to each use such that the maximum output voltage matches the maximum amplitude of the signal. This approach results in the best possible utilization of the DAC’s dynamic range for any given signal. Finally, a hybrid approach can be employed in which the potentiometer is set to a fixed value and the channel’s dynamic range is coarsely adjusted by a factor of 10 using the switch provided on the signal conditioning board depending on if a large signal (0.64–12.77 mV_pp_; 1 MΩ) or small signal (0.064–1.28 mV_pp_; 10 MΩ) is being reproduced. In this work, recalibration was performed for each biosignal tested to show the full capabilities of the system.

## 5. Case study: implantable device testing for aDBS

We then deployed NeuroDAC in a ‘real-world’ setting to explore adaptive deep brain stimulation (aDBS) algorithms using an investigational device (Summit RC+S Medtronic, Minneapolis, MN, USA) currently deployed in an on-going human clinical study (NCT03582891) [44]. Two bench-top testing paradigms were performed: (1) playing idealized, artificially generated, local field potential (LFP) patterns and (2) playing real neural signals captured during chronic recordings in human subjects.

One buffered channel of the NeuroDAC device was connected to a differential input channel of the RC+S through a standard quadripolar extension DBS lead and a symmetric star load tissue-model circuit [45]. A stimulation output channel of RC+S was connected between the two remaining resistors of the star load to allow for emulation of simultaneous recording and stimulation paradigms [45]. Taking advantage of the differential input amplifiers on the RC+S, the symmetry of the tissue-model circuit helps maximize common mode rejection of line noise and stimulation artifact.

**Figure 9.**
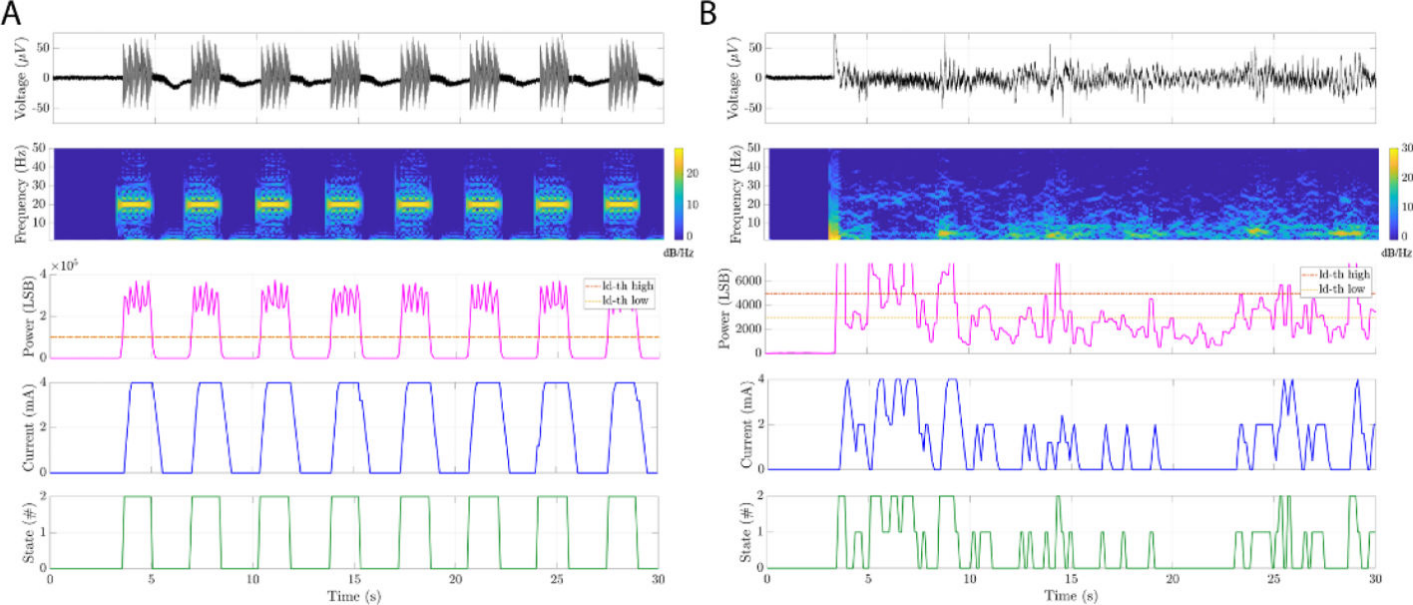
Examples of using NeuroDAC to perform bench-top testing of an adaptive neuromodulation device depicting two use cases: (A) an artificial LFP burst sequence at nominal frequency of 20 Hz and varying amplitude between 50 and 10 *μ*V and (B) playback of real neural data from GPi of a DBS patient. The panels show (from top to bottom), the time domain signal played by NeuroDAC and recorded by the RC+S, a spectrogram (0–50 Hz) of the signal, the output of the RC+S’s linear discriminant detector for in-band signal power, the stimulation current delivered by the RC+S, and the embedded algorithm’s decoded state for adaptive stimulation.

To test the basic functionality of the NeuroDAC along with the embedded detection algorithm on the RC+S, MATLAB (The Mathworks, Inc. Natick, MA, USA) was used to generate an artificial LFP signal which was played back through NeuroDAC (figure 9(A)). The 30 second simulated LFP waveform was comprised of eight 1.5 s, amplitude modulated waveforms, termed ‘long bursts’, with an interburst period of 2 seconds. Each ‘long burst’, in turn, was comprised of five 300 ms decreasing amplitude waveforms, termed ‘short bursts’. ‘Short burst’ amplitudes were varied between 50 and 10 *μ*V (steps of 10 *μ*V) with a nominal frequency of 20 Hz. These waveform parameters were chosen to approximate pathological beta band activity of the type that may be encountered *in vivo* [46]. As is shown in figure 9(A), the RC+S device was able to record and interpret the played-back signal, successfully change its internal state during periods of high ‘beta band activity’, and deliver stimulation.

Next, NeuroDAC was used to play back pre-recorded neural data from a patient with deep brain electrodes implanted in globus pallidus internus (GPi) and globus pallidus externus (GPe) (figure 9(B)). The recordings shown here are from the electrode contacts localized to the GPi. This protocol was approved by the University of San Fransisco Institutional Review Board (IRB protocol 18–24454). Data were recorded during an off-medication state, 10 days post-op, at a remote and supervised home visit by the research team. While replaying the signal using NeuroDAC, the RC+S device’s embedded firmware generated an internal estimation of the re-recorded signal’s power spectrum that could be used to update the device’s stimulation state [20]. A dual threshold detection approach was applied to selectively stimulate at the onset of beta band activity (16.6–26.37 Hz) to explore this feature as a potential biomarker of adaptive DBS. This band was identified during an *a priori* exploration of potential biomarkers from chronic home recordings during fluctuations in the patient’s movements. Again, figure 9(B) indicates that the RC+S was able to use the played-back signal to change state and deliver stimulation based on its beta band content. Note that the input waveform also contained periods of increased activity at lower frequencies, the RC+S was able to discriminate these and deliver stimulation in proportion to beta band activity.

## 6. Discussion

Biomedical devices capable of patient specific, real-time, closed loop operation have the potential to change the landscape of elecrophysiological therapeutics. However, to ensure patient safety and device efficacy, hardware must be fully validated, embedded algorithms must be efficiently prototyped, and new technologies must be exposed early in the development cycle to the real-world signals they will be required to manipulate. Bench-top testing with biosignal generators such as NeuroDAC represents a low-stakes environment for evaluating the behavior of new designs when presented with realistic elecrophysiological signals, their nuanced characteristics, and inherent noise that cannot be easily replicated with idealized ‘model’ data. Here we present an open-source low-cost option for researchers and manufacturers looking to build a biosignal waveform generator.

Using inexpensive off-the-shelf components based around the audio electronics industry and a simple, accessible signal conditioning circuit, the NeuroDAC presents a robust solution with minimum barriers to use. By expanding access to biosignal testing equipment, laboratories which may not otherwise have access to animal or clinical models can focus on embedded algorithm design and can test new ideas quickly and reliably. Users can adapt or modify the design easily to attain a desired feature set. Voltage divider values can be adjusted to resolve signals of different amplitudes and new analog conversion hardware could be implemented with a different frequency response, signal bandwidth, or hardware interface. Table 2 comprises a list of commercially available (at the time of this writing) audio DACs, other than the U-DAC8, which may be better suited to the needs of some users. Those devices featuring single ended outputs can be used directly with the NeuroDAC signal conditioning circuit with little to no modification. If standardized ‘benchmark’ datasets are published, large groups of researchers could compare their system’s performance with others, highlighting advancements in a controlled way. Some example use cases for NeuroDAC are for closed-loop algorithm development, analog front-end circuitry evaluation, signal processing/artifact rejection pipelines, tissue-electrode interface characterization, and troubleshooting of completed designs.

### 6.1. Limitations

Although NeuroDAC allows for playback of up to eight independent waveforms, some device testing may require a greater number of outputs. In these cases, it is possible to run multiple NeuroDAC devices in parallel, but future versions of NeuroDAC may support higher channel counts. Additionally, calibrating NeuroDAC requires manual adjustment of analog components, while simple and flexible this is a potential source of error in reproducing a signal. A more complex arrangement of digital potentiometers or a switched array of discrete resistors would allow for programmatic control over the device’s calibration and make for more consistent results. While most of the biosignals tested using NeuroDAC had excellent cross correlation coefficients of ≥0.95, the intracranial extracellular neural activity had a lower value of 0.83. It is possible that this discrepancy was caused by the high frequency content of this signal relative to the others. As depicted in figure 6, there is a phase shift in the frequency response above ~1 kHz. Slight distortion in the action potential waveforms may have resulted in a lower cross correlation coefficient in this case. Currently, the NeuroDAC design uses single ended analog outputs. Adding optional differential outputs would help eliminate common mode noise in those devices equipped with differential input analog front-ends [33]. Noise coupling should be carefully considered when setting up any electrophysiological testing environment. The BNC connectors used by NeuroDAC are ubiquitous in most laboratory settings but result in the use of long cables that are susceptible to noise. By building in a battery power supply and supporting common electrophysiology connectors such as Omnetics (Omnetics Connector Corporation, Minneapolis, MN, USA), Cere-Port (Blackrock Microsystems, Salt Lake City, UT, USA), or DIN 42802 IEC 60601 compliant ‘touch-proof’ connectors, future versions of NeuroDAC would minimize potential sources of external noise.

## 7. Conclusion

We present NeuroDAC, an open-source, low-cost option for building a biosignal waveform generator using a commercially available audio DAC and a simple circuit for signal conditioning. The full system architecture and usage procedures are documented making it easy to modify the design. We demonstrate the noise, frequency response, and practical performance of the device’s current implementation; as well as provide a real-world example using NeuroDAC to perform bench-top testing of a closed-loop neuromodulation device. NeuroDAC provides eight fully independent channels for unique signal playback and uses convenient standard USB communication and drivers to stream data directly from a host computer. Researchers can play back ‘ideal’ test signals or previously acquired or downloaded biosignal data that fully recreates the details of a real-world electrophysiological recording. Such a device is useful for bench-top testing and validation of biomedical devices such as closed-loop implants including cardiac pacing devices [8, 10, 16] and adaptive neuromodulation devices [1–3, 6, 9, 20, 21]. New algorithms can be tested on standard sets of data before they are deployed in a clinical setting. We believe convenient access to such a device will enable rapid iteration and prototyping of new therapeutic tools in a broad range of biomedical and neurological domains.

## 8. Open source

The authors have made the schematics for the NeuroDAC signal conditioning circuit and example code for using the device with both MATLAB and Python programming languages available under the MIT license in a public Github repository (https://github.com/neuromotion/neurodac). Please see DOI: 10.5281/zenodo.4069972

## Figures and Tables

**Table 1.** Comparison of commercial biosignal generator options.

Device	Headstage Tester Unit [28]	Digital Neural Signs Simulator [24]	ProSim 8 Vital Signal Simulator [29,30]	33 210A [31,32]	Haci, 2017 [27]	This Work
Manufacturer	Plexon	Blackrock Microsystems	Fluke	Keysight	N/A	N/A
Signal type	Arbitrary (continuous)	Neural, stepwise chirp	ECG (various)^3^	arbitrary (<8k points), sine, square, ramp, pulse, white noise	arbitrary (pre-loaded SD card)	arbitrary (continuous)
Number of channels	8–128	4–128	12-lead ECG	1	32	8
Number of simultaneous signals	1	1 (with phase offset on different channels)	1	1	32	8
Adjustable dynamic range	No	No	yes	yes	no	yes
Signal amplitude	Input divided— 1:1000^1^ or 1:10 000^2^	Not specified	0.05 mV-5 mV^4^	20 mV_*pp*_–20 V_*pp*_	141 mV_*pp*_	64 uV_*pp*_–12.78 uV_*pp*_–
Buffered output	No	Not specified	not specified	50 ohm output	no (4.5 kOhm)	yes
Inputs	Audio connector, test pins	N/A	USB device, USB host, wireless	USB, LAN, GPIB	SD card	USB
Outputs	Omnetics or Samtec	Omnetics, Samtec, header connector, Blackrock pedestal	universal ECG jacks	BNC	D-Sub	BNC
Cost	Not specified	$1250	$7,090.00	$1,433	$300 (estim.)	$450 (approx.)

1 Audio jack input.

2 Test pin input.

3 Other unrelated biosignals: respiration, temperature, IBP, cardiac output, NIBP, SpO2, rainbow multi-wavelength waveforms.

4 Amplitude from given to peak of R wave, based on reference Lead II (other leads vary from 24% to 120% of this value).

**Table 2.** Comparison of commercially available audio DACs that may be used to construct NeuroDAC as alternatives to the U-DAC8.

Device	D1 [47]	DACMAGIC 100 [48]	Modius [49]	E38 MKII [50]	U-DAC8 (this work) [38]
Manufacturer	Audioengine	Cambridge Audio	Schiit	exaSound	MiniDSP
Resolution (bits)	24	24	24	32^4^	24
Sample rate (kHz)	96^1^	192	192	384^4^	192
High pass filter (Hz)	10	20	20	DC	1
Line level output (V_rms_)	2	2.3	2^2^ or 4^3^	4.2	2
Channel number	2	2	2	8	8
Output type	Single ended	Single ended	Single ended or differential	Differential	single ended
Interface	USB, optical	USB, optical, coaxial	USB, optical, coaxial, AES	USB, optical, coaxial	USB
Chipset	AK4396	WM8742	AKM4113	ES9038PRO	AK4440
Cost	$169.00	$199.99	$199.00	$4475.00	$255.00

1 USB input.

2 Single ended output.

3 Differential output.

4 USB PCM input.
